# Genome-wide investigation reveals pathogen-specific and shared signatures in the response of *Caenorhabditis elegans *to infection

**DOI:** 10.1186/gb-2007-8-9-r194

**Published:** 2007-09-17

**Authors:** Daniel Wong, Daphne Bazopoulou, Nathalie Pujol, Nektarios Tavernarakis, Jonathan J Ewbank

**Affiliations:** 1 Centre d'Immunologie de Marseille-Luminy, Université de la Méditerranée, Case 906, 13288 Marseille Cedex 9, France; 2 Institut National de la Santé et de la Recherche Médicale, U631, 13288 Marseille, France; 3 Centre National de la Recherche Scientifique, UMR6102, 13288 Marseille, France; 4 Institute of Molecular Biology and Biotechnology, Foundation for Research and Technology, Heraklion 71110, Crete, Greece

## Abstract

**Background:**

There are striking similarities between the innate immune systems of invertebrates and vertebrates. *Caenorhabditis elegans *is increasingly used as a model for the study of innate immunity. Evidence is accumulating that *C. elegans *mounts distinct responses to different pathogens, but the true extent of this specificity is unclear. Here, we employ direct comparative genomic analyses to explore the nature of the host immune response.

**Results:**

Using whole-genome microarrays representing 20,334 genes, we analyzed the transcriptional response of *C. elegans *to four bacterial pathogens. Different bacteria provoke pathogen-specific signatures within the host, involving differential regulation of 3.5-5% of all genes. These include genes that encode potential pathogen-recognition and antimicrobial proteins. Additionally, variance analysis revealed a robust signature shared by the pathogens, involving 22 genes associated with proteolysis, cell death and stress responses. The expression of these genes, including those that mediate necrosis, is similarly altered following infection with three bacterial pathogens. We show that necrosis aggravates pathogenesis and accelerates the death of the host.

**Conclusion:**

Our results suggest that in *C. elegans*, different infections trigger both specific responses and responses shared by several pathogens, involving immune defense genes. The response shared by pathogens involves necrotic cell death, which has been associated with infection in humans. Our results are the first indication that necrosis is important for disease susceptibility in *C. elegans*. This opens the way for detailed study of the means by which certain bacteria exploit conserved elements of host cell-death machinery to increase their effective virulence.

## Abstract 2

Microarray analysis of the transcriptional response of C. elegans to four bacterial pathogens revealed that different infections trigger responses, some of which are common to all four pathogens, such as necrotic cell death, which has been associated with infection in humans.

## Background

Mammals defend themselves from infection via two inter-dependent types of immunity: innate and adaptive. Innate immune mechanisms represent front-line protection against pathogens and instruct the subsequent adaptive response. One of the principal attributes of the adaptive immune system is its remarkable specificity, based on somatic gene rearrangement and hypermutation leading to an extremely large repertoire of T- and B-cell receptors and antibodies. While such adaptive immunity is restricted to jawed vertebrates, invertebrates rely on their innate immune defenses. Until recently, these were generally considered to be relatively non-specific. For example, insects were known to mount distinct responses to different broad classes of pathogens (fungi, Gram-negative and Gram-positive bacteria) but assumed not to have pathogen-specific defense mechanisms [1]. There is, however, increasing evidence to suggest that the innate immune system may confer specific protection to the host even in invertebrates. For example, in insects, alternative splicing gives rise to thousands of distinct isoforms of the Dscam protein, a homolog of the human DSCAM (Down syndrome cell adhesion molecule) that has been proposed to be involved in pathogen recognition [2]. Different pathogens appear to stimulate the production of different subsets of Dscam isoforms and there is even the suggestion from studies with mosquitoes that isoforms preferentially bind the pathogen that induces their production [3]. Very recently, it has been shown that inoculation of *Drosophila melanogaster *with *Streptococcus pneumoniae *specifically protects against a subsequent challenge with this pathogen, but not against other bacterial species [4].

Nematode worms, such as *Caenorhabditis elegans*, are exposed to many pathogens in their natural environment and are expected to have evolved efficient defense mechanisms to fight infection. In the laboratory, *C. elegans *is cultured on an essentially non-pathogenic strain of *Escherichia coli*. This can easily be substituted with a pathogenic bacterium, readily allowing analysis of bacterial virulence mechanisms and host defenses. *C. elegans *has been used for the past few years as a model host for the study of the molecular basis of innate defenses, but compared to *D. melanogaster*, these studies are still very much in their infancy [5,6]. Nevertheless, using genetically diverse natural isolates of *C. elegans *and the bacterial pathogen *Serratia marcescens*, it has been shown that there is significant variation in host susceptibility and significant strain- and genotype-specific interactions between the two species [7]. Additionally, the transcriptional response of *C. elegans *to a number of different bacterial pathogens has been determined [8-11]. Given the relatively small overlap between the sets of genes identified as being transcriptionally regulated following infection with different pathogens, the combined results suggest a substantial degree of specificity in the innate immune response of *C. elegans*. One important caveat, however, is that these results were obtained in different laboratories using different microarray platforms. Indeed, as discussed further below, a comparison of two different studies both using *Pseudomonas aeruginosa *[10,11] revealed substantial differences in the apparent host response. This may reflect the known limitations of microarrays that have been well documented [12,13].

To investigate the specificity of the transcriptional response of *C. elegans *to infection, we have carried out a comparative microarray study at a fixed time-point using one Gram-positive and three Gram-negative bacterial pathogens. Their pathogenicity against *C. elegans *has been characterized previously [14-16]. Our analyses suggest that distinct pathogens provoke unique transcriptional signatures in the host, while at the same time they revealed a common, pathogen-shared response to infection. One prominent group of genes found within the pathogen-shared response was aspartyl proteases. These have diverse biological roles, including an important function in necrosis [17]. Consistent with this, we observed that bacterial infection was indeed associated with extensive necrotic cell death in the nematode intestine. Furthermore, using fluorescent reporter genes, we confirmed that aspartyl proteases implicated in necrosis are up-regulated during infection. In contrast to programmed cell death or apoptosis, necrosis is induced by environmental insults [18]. In many species, apoptosis serves a protective function, limiting pathogen proliferation [19]. Post-embryonic apoptosis in *C. elegans *occurs only in the somatic cells of larvae during early development, prior to the third larval (L3) stage, and in the germline of adult animals [20]. Germline apoptosis has been shown to mediate an increased resistance to *Salmonella *infection in *C. elegans *[21]. To address the question of whether necrosis observed in the adult soma during infection has a protective role, we analyzed the survival of necrosis-deficient mutants. We found that these animals were significantly more resistant to infection than wild-type worms, suggesting that necrosis is an integral and deleterious part of the infection-induced pathology. Since bacteria exploit conserved elements of the host's cell death machinery to increase their effective virulence, these results may provide insights into host-pathogen interactions in higher species.

## Results

### Exploratory analyses of host response to infection

To determine the degree of specificity in the response of *C. elegans *to bacterial infection, we carried out a whole-genome, comparative analysis of worms infected with one Gram-positive and three Gram-negative bacterial pathogens using long-oligo microarrays. We first looked at the response to *S. marcescens *and found less than a 2% overlap between the genes identified as being up-regulated by *S. marcescens *in this study (supplementary Table 1a in Additional data file 3) and a previous investigation, which employed a different microarray platform based on nylon cDNA filters with partial genome coverage [8]. This underlines the difficulty in making direct comparisons between studies employing different experimental designs.

Studies with *C. elegans *generally use worms cultured on the standard nematode growth medium (NGM) agar. On the other hand, the Gram-positive bacterium *Enterococcus faecalis *is most pathogenic when cultured on a rich medium (brain heart infusion (BHI) agar) [15]. To eliminate possible effects of the medium on nematode physiology, we wished to carry out all infections on worms grown on NGM agar. We determined that *E. faecalis *was still pathogenic to *C. elegans *when grown on NGM agar, if pre-cultured in liquid BHI medium (supplementary Figure 1 in Additional data file 1), and adopted this protocol for our analyses.

**Figure 1:**
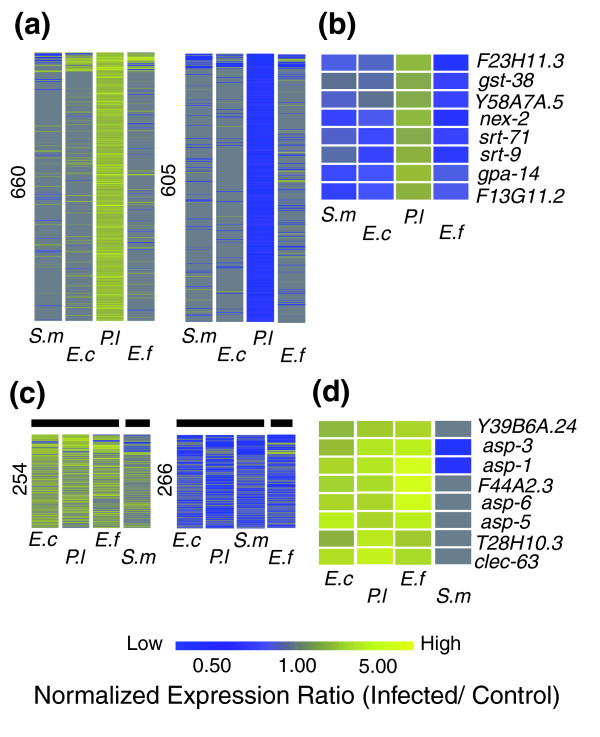
Comparison of host gene expression profiles following infection with different pathogens. Expression levels are indicated by a color scale and represent normalized differences between infected and control animals. Grey denotes genes not considered to be differentially regulated under that condition. The numbers on the vertical axis correspond to differentially regulated genes. Each column shows the expression levels of individual genes (represented as rows) following infection by the pathogens as indicated on the horizontal axis (*S. m*, *S. marcescens*; *E. f*, *E. faecalis*; *E. c*, *E. carotovora*; *P. l*, *P. luminescens*). **(a) **Genes differentially regulated in an infection with *P. luminescens *and their comparative expression levels with other pathogens. **(b) **Genes defining a pathogen-specific signature specifically up-regulated with *P. luminescens *infection. **(c) **Groupings, as indicated by the horizontal bars, formed after clustering using non-redundant sets of genes that were up- and down-regulated by at least two pathogens (trees not shown). **(d) **Genes commonly up-regulated following *E. carotovora*, *E. faecalis *and *P. luminescens *infections.

Comparing the levels of expression for genes that were up- or down-regulated at a single time point by each individual bacterial pathogen (*S. marcescens*; *E. faecalis*; *Erwinia carotovora*; *Photorhabdus luminescens*), we observed expression profiles that were characteristically unique, or 'pathogen-specific signatures'. For example, the majority of genes with expression levels altered in one direction following infection by *P. luminescens *were either unchanged or responded differently in infections with other pathogens (Figure 1a,b; supplementary Table 1a,b in Additional data file 3). Thus, 24 h post-infection, *C. elegans *is clearly capable of mounting a response that is principally different for each of the pathogens used in this study. From non-redundant groups of 2,171 genes up-regulated and 2,025 genes down-regulated after infection with at least one pathogen, only 254 and 266 genes, respectively, were identified to be commonly regulated by more than one pathogen (supplementary Table 1c in Additional data file 3). These comparatively small numbers reinforce the notion of pathogen-specific responses, while at the same time suggesting that host responses to different pathogens have common facets. To examine this further, we performed clustering analyses with both the commonly up- and down-regulated genes. In both cases, groupings composed of genes responding similarly to different pathogens were observed (Figure 1c). Surprisingly, the response to the Gram-positive bacterium, *E. faecalis*, overlapped to a greater extent with those provoked by the Gram-negative bacteria *P. luminescens *and *E. carotovora *than did the response provoked by a third Gram-negative bacterium, *S. marcescens*. Thus, for example, one grouping was identified for genes with altered expression following infection with the first three bacteria, to the exclusion of *S. marcescens *(Figure 1d). Overall, highest similarity existed between the genes whose expression was altered following infection with *E. carotovora *and *P. luminescens*.

The large numbers of genes identified as being transcriptionally regulated upon infection represents a challenge for meaningful interpretation. In our study this problem was further compounded by the inclusion of multiple pathogens, which as a consequence, required the analysis of diverse datasets. The use of Gene Class Testing [22] to identify functional associations can, however, help in the identification of biologically relevant themes. We therefore used the freely available Expression Analysis Systematic Explorer (EASE) [23] to identify gene classes significantly over-represented among genes regulated as a consequence of infection. In our analyses, we looked at gene classes derived using Gene Ontology, euKaryotic Orthologous Groups and functional information from published experiments using *C. elegans *(see Materials and methods). Biological themes were formed via the grouping of gene classes in an *ad hoc *fashion, with all members of a group having similar biological functions. For example, the 'infection-related response' class includes genes described in published studies as being up- or down-regulated by infection, together with any structurally homologous genes.

**Figure 2:**
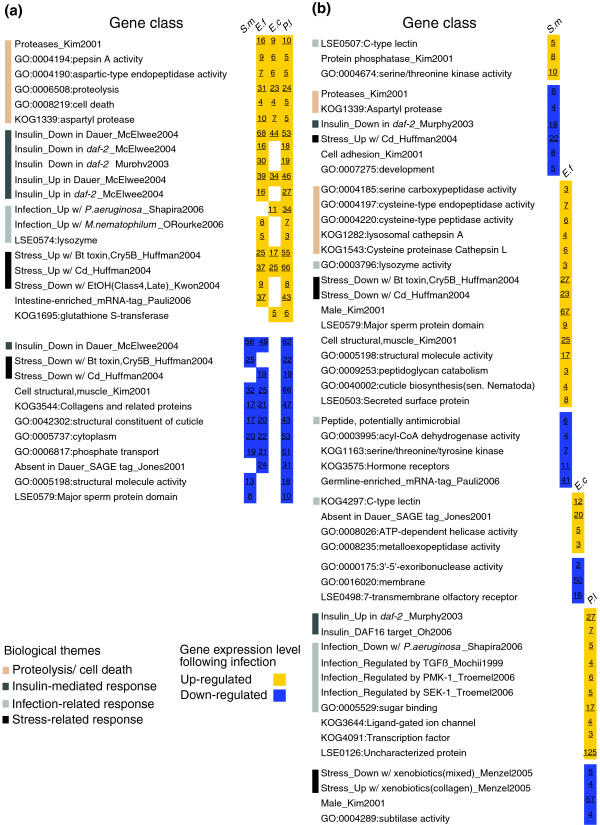
Gene classes within gene expression profiles identified using EASE. Significantly enriched gene classes (*p *value < 0.05) with genes that were differentially regulated following infection with the four pathogens (*S. m*, *S. marcescens*; *E. f*, *E. faecalis*; *E. c*, *E. carotovora*; *P. l*, *P. luminescens*). Expression profiles were either **(a) **similar, or **(b) **different across pathogens. Numbers shown indicate the number of genes significant in that gene class, whilst relevant biological themes are indicated with lines in different colors.

With EASE we identified two major groupings of gene classes. The first, termed 'pathogen-shared', is composed of gene classes identified across infections with different pathogens (Figure 2a; supplementary Table 2a in Additional data file 3). These include classes shared by genes with similar expression profiles in *E. faecalis*, *E. carotovora *and *P. luminescens *infections and that can be further associated with proteolysis, cell death, insulin signaling and stress responses. Other gene classes shared by *E. faecalis *and *P. luminescens *include lysozymes, genes expressed in the intestine and genes implicated in the response to infection with *Microbacterium nematophilum*, a Gram-positive nematode-specific pathogen [9]. There was similarly an over-representation of genes up-regulated following infections with *E. carotovora *and *P. luminescens *that are associated with infection by another Gram-negative pathogen, *P. aeruginosa *[11]. A second grouping defined the 'pathogen-specific' responses (Figure 2b; supplementary Table 2b in Additional data file 3). For example, only *E. faecalis *infection was associated with a significant down-regulation of hormone receptors, while *P. luminescens *infection involved a significant elevation of the proportion of genes described to be under the control of p38 MAPK and TGF-β signaling pathways [10,24]. Biological themes associated with host response to adverse conditions, including infection, can be found within both the pathogen-specific and pathogen-shared groupings (Figure 2). Thus, as further discussed below, clustering analysis of gene expression and gene class testing are both consistent with the notion that the response of *C. elegans *to infection can be defined by two biologically relevant signatures, one being pathogen-shared and the other, pathogen-specific.

### Statistical testing of gene expression

While fold change measurements are conceptually useful when performing exploratory analyses, they lack known and controllable long-range error rates [22]. We therefore performed complementary analyses in which exploratory findings using fold change-derived data were combined with results obtained using two established statistical tools, MAANOVA and BRB-ArrayTools (see Materials and methods). With the two exploratory analyses, a grouping of host-responses observed following infection with *E. carotovora*, *E. faecalis *and *P. luminescens *was the most consistent (Figures 1c and 2a). We therefore used MAANOVA and BRB-ArrayTools on microarray data obtained with these three pathogens to investigate further the nature of this apparent pathogen-shared host-response. We identified a total of 22 high-confidence genes with significant differences in expression between control animals and animals infected with the three pathogens (Table 1; supplementary Table 3a in Additional data file 3). Prominent among these 'common response genes' is *lys-1*, which was one of the first infection-inducible genes to be identified in *C. elegans *[8]. Following the demonstration that it was up-regulated by *S. marcescens *infection, *lys-1 *has also been shown to be part of the response of the worm to *P. aeruginosa *[11]. The list also includes a gene that encodes a lipase, a class of protein important in the response to *S. marcescens *[8] and *M. nematophilum *[9], as well as a saposin-encoding gene. All the corresponding proteins are expected to have antimicrobial activity and, therefore, to contribute directly to defense [25,26]. Other genes correspond to a C-type lectin (*clec-63)*, a putative LPS-binding protein (*F44A2.3*), and proteins containing Complement Uegf Bmp1 (CUB) and von Willebrand Factor (vWF) domains and vWF, epidermal growth factor (EGF) and lectin domains, respectively; all of these could be involved in pathogen recognition [25,26]. Members of the largest class of genes, however, encode aspartyl proteases not previously associated with the response to infection in *C. elegans*.

**Table 1:** Common response genes in the pathogen-shared host response

			Microarray data					
			
						Set of three datasets (*E. f*, *E. c *and *P. l*)		
						
						BRB-ArrayTools		MAANOVA
							
			Fold change (infected/control)			(Infected/control)		
						
Sequence name	Gene name	Brief description	*E. f*	*E. c*	*P. l*		*p *value	*p *value
**Up-regulated genes**								
T28H10.3		Asparaginyl peptidases	1.67	1.29	2.43	1.67	3.47E-05	1.17E-02
Y39B6A.20	*asp-1*	Aspartyl protease	3.54	1.80	2.17	2.09	2.06E-05	<1.00E-07
H22K11.1	*asp-3*	Aspartyl protease	2.59	1.47	2.29	1.96	4.80E-06	<1.00E-07
F21F8.3	*asp-5*	Aspartyl protease	2.53	2.48	1.86	2.06	2.83E-05	<1.00E-07
F21F8.7	*asp-6*	Aspartyl protease	2.96	1.89	1.88	-	-	<1.00E-07
Y39B6A.24		Aspartyl protease	1.84	1.40	1.62	1.59	1.21E-05	<1.00E-07
F44A2.3		BPI/LBP/CETP family protein	3.43	1.73	2.03	2.29	5.00E-07	2.35E-03
F35C5.6	*clec-63*	C-lectin	1.95	2.05	2.62	2.23	1.00E-07	<1.00E-07
Y37E3.15a	*npp-13*	Cullin	1.89	-	1.57	1.62	5.30E-06	-
T21H3.1		Lipase	1.38	1.99	1.89	1.85	8.00E-07	<1.00E-07
Y22F5A.4	*lys-1*	Lysozyme	1.33	1.30	1.81	-	-	4.82E-02
F59A1.6		Saposin A	1.92	1.82	1.92	1.78	2.60E-05	-
W02D7.8		Uncharacterized, nematode-specific	-	1.46	2.20	1.64	3.49E-05	-
ZK1320.3		Uncharacterized, nematode-specific	1.51	1.85	1.63	1.54	5.70E-06	-
F28B4.3		von Willebrand factor type A	2.28	-	2.09	-	-	4.14E-02
K06G5.1		von Willebrand factor type A	1.51	1.27	1.91	-	-	2.55E-02
								
**Down-regulated genes**								
C55B7.4a	*acdh-1*	Acyl-CoA dehydrogenase	0.33	0.47	0.35	0.35	<1.00E-07	<1.00E-07
C17C3.12b	*acdh-2*	Acyl-CoA dehydrogenase	0.59	0.54	0.52	0.54	1.00E-07	<1.00E-07
Y38F1A.6		Alcohol dehydrogenase, class IV	0.59	0.54	0.53	0.55	8.00E-07	<1.00E-07
T05G5.6	*ech-6*	Enoyl-CoA hydratase	0.55	0.62	0.49	0.62	3.00E-06	<1.00E-07
K02F2.2		S-adenosylhomocysteine hydrolase	0.67	0.69	-	0.70	8.20E-06	4.69E-03
F54D11.1	*pmt-2*	SAM-dependent methyltransferases	0.67	0.68	0.66	0.69	1.13E-05	-

*E. c*, *E. carotovora*; *E. f*, *E. faecalis*; *P. l*, *P. luminescens*.

Neither up- nor down-regulated genes exhibited any substantial genomic clustering of the type described for genes involved in the response to *M. nematophilum *infection [9]. With regards to down-regulated genes within the pathogen-shared response identified in this study, they are all seemingly metabolism-related; a similar phenomenon has been previously described in worms infected with *M. nematophilum *[9].

### Validation of common response genes by quantitative real-time PCR

**Figure 3:**
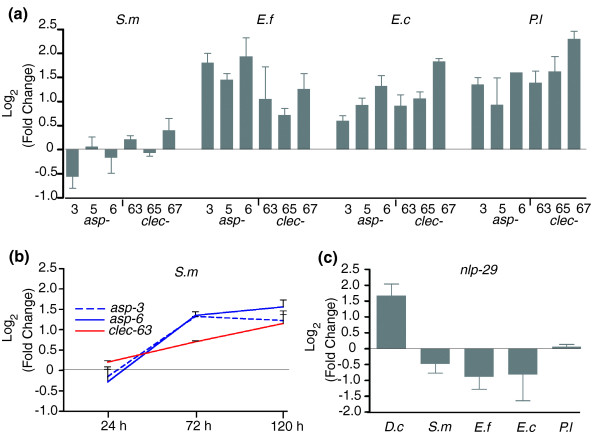
qRT-PCR analyses. **(a) **Expression levels of common response genes representing two gene families were measured and data reported as mean difference between infected and control animals following infection with the four pathogens (*S. m*, *S. marcescens*; *E. f*, *E. faecalis*; *E. c*, *E. carotovora*; *P. l*, *P. luminescens*). **(b) **The expression levels of *asp-3*, *asp-6 *and *clec-63 *were followed over a period of five days in *C. elegans *infected with *S. marcescens*; data reported as mean difference between infected and control animals. Bars represent standard errors (at least two independent measurements). **(c) **The antimicrobial gene *nlp-29 *responds specifically to fungal infection. The expression levels of *nlp-29 *were measured following infection with the fungal pathogen (*D. c*, *D. coniospora*) and the four bacterial pathogens. Data are reported as mean difference between infected and control animals. Bars represent standard errors (three independent measurements).

To validate these results, we examined in more detail the regulation of three *asp *genes encoding aspartyl proteases, as well as a C-lectin, encoded by *clec-63*, using quantitative real time-PCR (qRT-PCR). Since only a small number of common response genes was identified during statistical testing, we also looked at the expression of two other *clec *genes, one being *clec-65*, the genomic neighbor of *clec-63*, and the other *clec-67*, reported to be induced by *M. nematophilum *[9]. At 24 h, all six genes showed a marked up-regulation following infection by *E. faecalis*, *E. carotovora *and *P. luminescens*, whereas they did not show a substantial change in expression following *S. marcescens *infection (Figure 3a). We hypothesized that this result could be a consequence of the different pathogenicities of the bacteria. To investigate this, we carried out a time course study over a period of five days, using qRT-PCR to follow relative expression levels of *asp-3*, *asp-6 *and *clec-63 *in worms infected by *S. marcescens*. The expression levels of these three genes indeed increased over this period (Figure 3b), suggesting that their induction is linked to pathogenesis more than to pathogen recognition.

### Common response gene transcription is not altered by fungal infection

In contrast to the bacterial pathogens used in this study that infect *C. elegans *via the intestine, the fungus *Drechmeria coniospora *infects nematodes via the cuticle [27]. A comparison of the common response genes with those having an altered expression following infection with *D. coniospora*, determined under similar experimental conditions to those used in this study (Pujol *et al*., submitted), showed absolutely no overlap (results not shown). This clear distinction between bacterial and fungal infection was unexpected since we had previously reported, based on our results using cDNA microarrays, that the antimicrobial peptide gene *nlp-29 *was induced upon infection both by *S. marcescens *and *D. coniospora *[27]. This gene appeared not to be up-regulated, however, by any of the bacterial pathogens used in this study, including *S. marcescens*. When we assayed the level of *nlp-29 *expression in worms infected by the different pathogens using qRT-PCR, we found that only *D. coniospora *induced a substantial increase (Figure 3c). We recently found that *nlp-29 *is induced under conditions of high osmolarity (Pujol *et al*., submitted), including when plates used for culturing worms become drier after a few days storage. The age of plates was not a variable that was previously controlled, and we now believe this to be the most likely reason for having erroneously identified *nlp-29 *as a gene induced by *S. marcescens *infection. These results underline the fact that *C. elegans *mounts distinct responses to bacterial and fungal infection.

### Expression domains of common response genes

**Figure 4:**
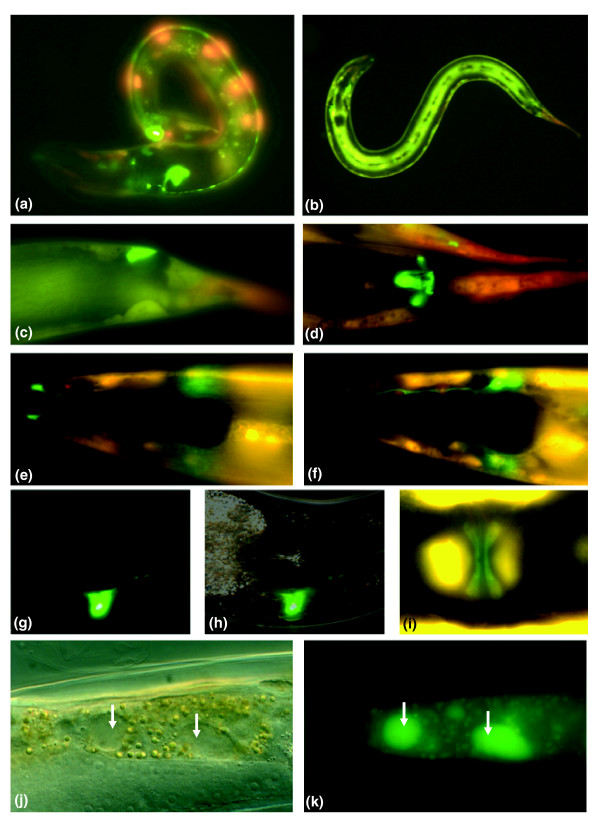
Expression domains of common response genes and symptoms associated with infection. p*F44A2.3*::GFP expression in the **(a) **ventral nerve-cord, **(b) **hypodermis, **(c-d) **P12.pa pre-anal cells, **(e-f) **glial amphid socket cells, **(g-h) **excretory duct cell and **(i) **vulE or uv1 cells. Red fluorescence comes from the p*col-12::dsRED *co-injection marker. In areas where both GFP and dsRED are expressed, yellow is observed. **(j,k) **Vacuoles (arrows) can be observed within intestinal cells of *P. luminescens*-infected adults (j), in which there is detectable expression of *asp-4*::GFP (k). Similar results were obtained with infected adults expressing *asp-3*::GFP. In contrast, no GFP expression or vacuolization was seen in the intestines of non-infected worms.

The response of *C. elegans *to infection by *S. marcescens *and *P. aeruginosa *involves predominantly genes expressed in the intestine [8,11]. Information regarding the expression patterns for 19 of the 22 common response genes differentially regulated after infections with *E. faecalis*, *E. carotovora *and *P. luminescens *is available (supplementary Table 3a in Additional data file 3). Of these, 16 are expressed in the intestine of the adult animal. Examination of their proximal promoter regions using BioProspector [28] revealed GATA motifs in 43% of these genes (supplementary Table 3a in Additional data file 3), consistent with similar findings from a recent study [11]. Two other genes, *npp-13 *and *K06G5.1*, are known to be expressed in the gonad. By *in situ *hybridization, the remaining gene, *F44A2.3*, is reported to show weak but specific expression at the vulva and in the head. This gene also attracted our attention as it encodes a protein containing a lipopolysaccharide-binding protein (LBP)/bactericidal permeability-increasing protein (BPI)/cholesteryl ester transfer protein carboxy-terminal domain (Pfam accession number PF02886), associated with bacterial recognition or killing in many species [29,30]. We determined its expression pattern by generating transgenic strains carrying green fluorescence protein (GFP) under the control of the *F44A2.3 *promoter. We observed high levels of constitutive GFP expression in the pre-anal, vulval, hypodermal, glial amphid socket and excretory duct cells of the adult animal (Figure 4a-i). Upon infection of worms carrying the reporter gene with *E. carotovora *or *P. luminescens*, there was no perceptible change in the level of GFP expression at 24, 48 or 72 h post-infection (results not shown). Similarly, these two pathogens caused no discernable induction of GFP expression at any time up till 72 h post-infection in strains carrying 5 other transcriptional reporter genes (*asp-5 *and *-6*, *clec-63*, *-65 *and *-67*; results not shown). Thus, based on the genes tested, we were unable to identify robust *in vivo *reporters for the response to bacterial infection. The cells that expressed *pF44A2.3*::GFP are in privileged sites, in contact with the external environment, hinting at a potential front-line role for *F44A2.3 *in pathogen recognition. We addressed any potential role in resistance to infection by inactivating its expression by RNAi, but did not see any significant effect on survival (supplementary Figure 2 in Additional data file 1).

### Necrosis aggravates infection-associated pathology

In contrast to the reporter genes listed above, we observed a clear and reproducible induction of expression of the *asp-3 *and *-4 *reporter genes. In the absence of infection, virtually no GFP was detectable, while after exposure to *E. carotovora *or *P. luminescens *there was an accumulation of GFP within large vacuoles formed in the intestine (Figure 4j-k). We observed a qualitatively similar induction of reporter gene expression following infection with *E. faecalis *but of a lower magnitude (results not shown).

When the *asp-4*::GFP reporter was transferred by mating into *pmk-1(km25) *or *dbl-1(nk3) *mutant backgrounds, we observed an induction of GFP expression following infection with *E. carotovora *that was similar to that seen in wild-type worms (results not shown). The two mutants respectively affect the p38 MAPK and TGF-β pathways, important for resistance to bacterial infection. Thus, these results suggest that infection-induced expression of ASP-4 is independent of the two pathways.

**Figure 5:**
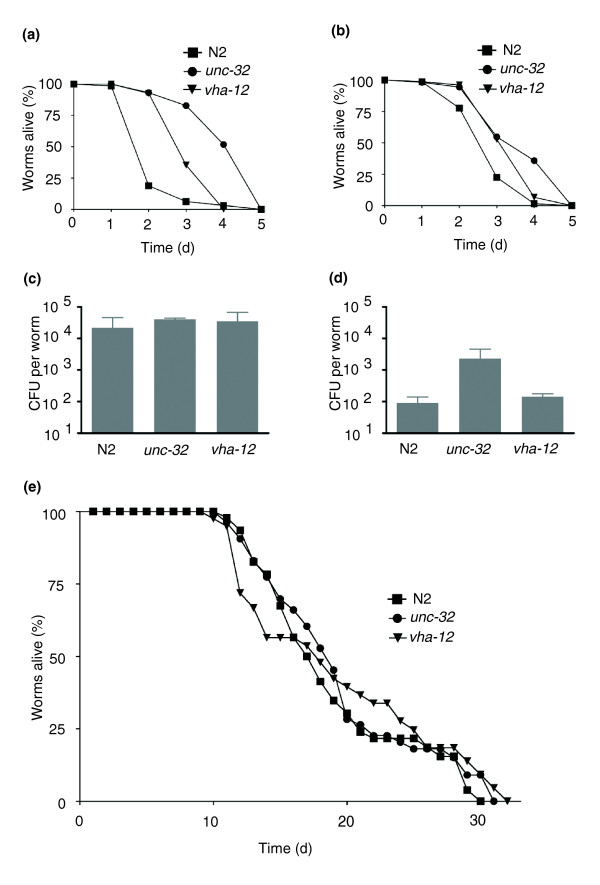
Suppression of necrosis increases resistance of worms to infection. Both *vha-12(n2915) *and *unc-32(e189) *are associated with a defect in vacuolar H^+^-ATPase activity and, consequently, reduced necrosis. Following infection with **(a) ***E. carotovora *and *E. carotovora ***(b) ***E. carotovora*, the differences between wild-type N2 and *vha-12(n2915) *or *unc-32(e189) *survival are highly significant (log-rank test, *p *value < 0.0001). Data shown are representative of three independent experiments. **(c,d) **Bacterial load in the intestines of wild-type and mutant *C. elegans *(indicated on the horizontal axes), after 24 h exposure to *E. carotovora *(c) and *P. luminescens *(d). The number of colony-forming units (CFU) per worm was measured and bars represent the standard errors from two independent experiments. **(e) **Life-span assays for the mutants *vha-12(n2915) *and *unc-32(e189) *and wild-type N2 on non-pathogenic OP50 *E. coli*. Differences between the three strains are not significant (log-rank test, *p *value > 0.05).

Both *asp-3 *and *-4 *have been specifically associated with the execution of necrotic cell death in *C. elegans *[17]. Indeed, inspection of worms during infection revealed the frequent incidence of necrotic cell death in the intestine, which is manifested by the vacuole-like appearance of cells (Figure 4j), not seen within the intestine of healthy animals. These dramatically swollen cells have distorted nuclei restricted in the periphery, a most prominent characteristic of necrotic cell death. Preliminary observations suggested that infection under different culture temperatures (25°C and 20°C) progresses similarly in terms of symptoms and *asp*::GFP reporter gene expression, except that at 25°C everything was more rapid. In subsequent experiments, we therefore conducted infections at 20°C to increase the temporal resolution. The appearance of necrosis follows the spatiotemporal progression of infection. The first tissue affected is the intestine, where vacuolated cells were observed around 24 h post-infection. After the second day of infection, the epidermis and the gonad become severely distorted and displayed similar necrotic vacuoles. This pattern of necrotic death, observed following infection with different pathogens, could be part of an inducible defense mechanism contributing to host survival, or a deleterious consequence of infection. To differentiate between these two possibilities, we assayed the resistance to infection of two necrosis-deficient *C. elegans *mutants, *vha-12(n2915) *and *unc-32(e189)*, that both affect V-ATPase activity [31,32]. The two mutants showed enhanced survival, relative to wild-type N2 worms in infections with *E. carotovora *(Figure 5a) and *P. luminescens *(Figure 5b). Given that these mutants display abnormal pharyngeal pumping, we were concerned that resistance might be the consequence of a reduced bacterial load. We therefore directly assayed the number of viable bacteria within worm intestines at 24 h post-infection. With *E. carotovora*, there was no difference between infected wild-type and mutant animals (Figure 5c), while for *P. luminescens*, *unc-32 *animals had a higher bacterial load (Figure 5d). Therefore, differences in bacterial accumulation are not correlated with resistance of the two mutants to infection. Certain mutants of the insulin/insulin growth factor signaling pathway, such as *daf-2*, exhibit increased pathogen resistance and longevity [33]. To examine whether *vha-12 *and *unc-32 *are more infection-resistant due to general effects in survival and ageing, we measured the longevity of these mutants on non-pathogenic *E. coli *and found that they had similar lifespans to wild-type animals (Figure 5e), consistent with previous findings [34]. We also observed that the induction of *asp-4*::GFP by *E. carotovora *and *P. luminescens *was unchanged in a *vha-12 *mutant background (supplementary Figure 3 in Additional data file 1). Thus, mutants that have a defect in intracellular organelle acidification are necrosis-deficient and exhibit a specific increase in their resistance to infection that appears to be independent of *asp-4 *activity.

## Discussion

In vertebrates, in addition to the highly specialized and specific mechanisms of the adaptive immune system, a first line of defense constituted by the innate immune system involves the recognition of different classes of pathogens via germline-encoded proteins such as the Toll-like receptors [35]. The degree to which invertebrates are also able to respond specifically to infection is a question of considerable interest [36]. In this study we investigated whether infection of *C. elegans *by taxonomically distinct bacterial pathogens provokes distinct changes in gene expression. A principal motivation for the study was the difficulty in drawing conclusions from comparisons between studies using different experimental designs. For example, of a total of 392 genes reported to be induced in worms infected with *P. aeruginosa *in two independent studies, less than 20% were found in both [10,11]. With regards to our own results, there was essentially no overlap between the genes or gene classes found to be up-regulated by *S. marcescens *in this and a previous study [8].

Through the use of exploratory analyses, we identified genes that are regulated differentially by the pathogens used in this study. Employing three biologically replicated datasets from synchronized populations at a single time-point and the computational methods described, a robust statistical significance could not be ascribed to changes in individual gene expression associated with the pathogen-specific responses. This is probably because the datasets for individual pathogens were relatively small and contained inherent experimental variation. Nevertheless, a strong trend emerged from the groups of non-overlapping genes that define these responses, and when combined with results from previous studies [8-11] strongly suggest that *C. elegans *is capable of mounting a distinct response to different bacterial pathogens.

In contrast to the above, with the use of these same statistical tools we were able to define a group of common response genes having similar expression profiles across infections with three different pathogens (Table 1). We consider this high-confidence group to be a minimum set, since it is possible that a more extensive study employing more replication in the experimental design, different time-points or changed for other parameters would reveal additional genes to be commonly regulated by multiple pathogens. Pathogens that vary considerably in their virulence and that provoke different symptoms were used. Therefore, in the context of this study, common response genes are potentially constituents of mechanisms underlying a pathogen-shared, host-response to different infections. Many of these genes have been functionally characterized as participating in the response of *C. elegans *to various forms of stress as well as to infection by bacterial pathogens. Specific examples include *lys-1 *and *clec-63*, a lysozyme and C-type lectin, respectively. Both the lysozyme and C-type lectin classes of genes are known to have roles in innate immunity [8,9]. The expression of *lys-1 *is also modulated by insulin signaling [37] and by a toxin-induced stress response [38]. Taken as a whole, this suggests that common response genes may be regulated not only as a direct result of infection, but also by other factors consequent upon infection.

On the other hand, common response genes are not induced by infection with the fungus *D. coniospora*. Indeed, the signature of gene transcription associated with fungal infection is completely different from that provoked by the four bacterial pathogens used in this study. As discussed above, the antimicrobial peptide gene, *nlp-29 *is induced only by *D. coniospora*. We had previously reported that a second antimicrobial peptide gene, *cnc-2*, was induced upon infection both by *S. marcescens *and *D. coniospora*, based on our results using cDNA microarrays [27]. *cnc-2 *was found to be up-regulated by *P. aeruginosa *infection [10] and suggested to be a 'general response gene'. Like *nlp-29*, *cnc-2 *appeared not to be up-regulated by any of the bacterial pathogens used in this study, nor in our hands by *P. aeruginosa *(CL Kurz, personal communication). Nor was *cnc-2 *induced by high osmolarity (OZugasti, personal communication). On the other hand, the structurally related gene *cnc-7 *is up-regulated under conditions of osmotic stress (T Lamitina, personal communication). The cDNA microarrays we used previously do not have a *cnc-7*-specific probe, but the sequence of the *cnc-7 *mRNA is >80% identical to that of *cnc-2*. Therefore, it is possible that dry plate conditions induced *cnc-7 *expression and cross-hybridization resulted in the erroneous detection of increased *cnc-2 *transcript levels.

As mentioned previously, the down-regulated common response genes identified in this study appear to have functions associated with general metabolism. For example, the genes that show the greatest down-regulation, *acdh-1 *and *-2*, encode acyl-CoA dehydrogenases involved in mitochondrial β-oxidation and the metabolism of glucose and fat. Their expression levels are also repressed upon starvation [39,40]. The modulation of their expression by pathogens could reflect a reduction in food uptake upon infection, or be part of a mechanism to control cellular resources and limit their availability to pathogens. The role that transcriptional repression plays in the innate immune response of *C. elegans *must be the subject of future studies.

Common response genes identified in this study include a grouping of seven genes associated with proteolysis and cell death, *asp-1*, *3*, *4*, *5 *and *6*, *T28H10.3 *and *Y39B6A.24*. With the exception of *Y39B6A.24*, all others are known to be expressed in the intestine (supplementary Table 3b in Additional data file 3). Using information from the Pfam database [41], all seven have been annotated as possessing a potential amino-terminal signal sequence. Interestingly, the remaining member of the aspartyl protease-encoding ASP family, ASP-2, which is not part of the pathogen-shared response, does not possess a comparable signal-sequence. While some aspartyl proteases within the cathepsin Esub-family are known to be secreted into the nematode intestine [42], experimental observations with full-length GFP fusions for ASP-3 and -4 indicate a predominantly lysosomal localization [17]. This suggests that the intracellular targeting of up-regulated proteases to lysosomes and perhaps other sub-cellular organelles, such as mitochondria, may be crucial for their proper functioning.

In *C. elegans*, necrosis is the best characterized type of non-apoptotic cell death [18]. Necrotic cell death is triggered by a variety of both extrinsic and intrinsic insults and is accompanied by characteristic morphological features. Our findings provide the first description of pathogen-induced necrosis in this model organism. While necrosis has been associated with infection in other metazoans, its role during infection remains unclear. Necrosis has been implicated in defensive or reparative roles following cellular damage, and necrotic cell death in tissues that have been compromised after vascular-occlusive injury triggers wound repair responses [43]. Successful pathogens overcome physical, cellular, and molecular barriers to colonize and acquire nutrients from their hosts [44]. In such interactions, it has been suggested that the cellular machinery of the host may in fact be exploited by viral and bacterial pathogens that induce necrotic cell death, resulting in damage to host tissue. For example, during *Shigella*-mediated infection, necrosis-associated inflammation is induced within intestinal epithelial cells of the host by the pathogen [45].

Our results suggest that in *C. elegans*, some experimental bacterial infections provoke a common program of gene regulation with consequences that include the promotion of necrosis in the intestine. Thus, these bacteria appear to exploit the necrotic machinery of *C. elegans *via a common host mechanism. While pathogen-induced necrosis might be protective for some infections, for the two bacteria tested, it appears to have no protective role and apparently hastens the demise of the host during the course of infection. Although there is increasing evidence for co-evolution between *C. elegans *and *S. marcescens *[7,46], and *E. carotovora*, *E. faecalis *and *P. luminescens *can be found in the soil [47-49], there is no reason to believe that the bacteria used in this study developed virulence mechanisms to induce necrosis specifically in *C. elegans*.

**Figure 6:**
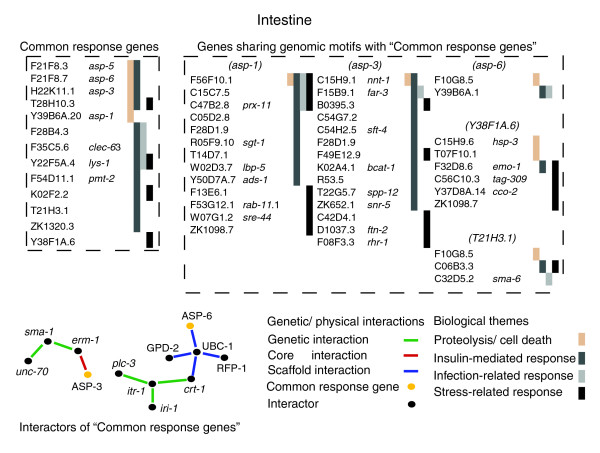
Modeling the molecular basis underlying an intestine-localized, pathogen-shared response to infection in *C. elegans*. Three major components make up the model; the common response genes identified directly in this study, genes associated with common response genes on the basis of shared DNA motifs, and interactors of the common response genes, either genetic (Wormbase) or physical (core or scaffold; InteractomeDB). Unambiguous evidence for expression in the intestine exists for all indicated genes. The relevant biological functions are shown in different colors.

In many cases, groups of genes that function together in the host response to pathogens or parasites share common regulation [11,50]. We sought to identify other genes that potentially function alongside common response genes within the intestine, but that were not identified for whatever reason as being transcriptionally regulated in this study. These include those having the potential for common transcriptional regulation. Unfortunately, there is still no simple relationship between transcriptional co-regulation and regulatory motifs [51]. Efforts are being made to this end, however, and data for regulatory motifs in *C. elegans *are available within the cis-Regulatory Element Database (cisRED) [52]. Relevant information could be obtained for only five common response genes expressed in the intestine (supplementary Table 4a in Additional data file 3). These are associated via shared, predicted motif groups with a number of other intestinally expressed genes (Figure 6; supplementary Table 4b in Additional data file 3). All five common response genes are associated with biological themes relevant to infection (see Results) and we observed similar associations with a number of the genes having shared genomic motifs (Figure 6; supplementary Table 4c in Additional data file 3). We postulate that these genes, associated with common response genes on the dual basis of shared motifs, found within genomic regions conserved across closely related species, and functional relevance, may potentially be intestine-localized components of a pathogen-shared response.

We also took advantage of published interaction data from InteractomeDB [53,54] and WormBase [55], to identify other genes and proteins that could potentially function alongside common response genes within the intestine. Of all common response genes expressed in the intestine, relevant interaction networks could be established only for *asp-3 *and *asp-6 *(Figure 6; supplementary Table 4d in Additional data file 3). With the exception of the interaction between ERM-1 and ASP-3 that was identified in a large-scale study, all other interactions shown have additional evidence obtained via small-scale studies. ERM-1 appears to be primarily involved in the maintenance of intestinal cell integrity; abrogation of *erm-1 *function by RNAi provokes distortion of the intestinal lumen in the adult animal [56]. In the case of *itr-1 *and *crt-1*, both have been implicated in the control of necrotic cell death [57] via regulation of intracellular calcium [18]. It follows that in the context of an interaction-network, their association with the common response gene *asp-6 *may be an indication of their involvement in intestinal cell necrosis provoked by infection. Such a possibility awaits experimental verification.

## Conclusion

This study has revealed that the infection of *C. elegans *with different bacterial pathogens can be characterized by a host response that is both pathogen-specific and pathogen-shared in nature. Unique gene expression profiles, which define the pathogen-specific responses to infection, are associated with common biological functions relevant in the context of host innate immunity. Necrosis, induced by different bacteria in the pathogen-shared response to infection, has a common basis at the molecular level, appears to have no obvious protective-role and its suppression increases host resistance. Consequently, targeting molecular components to prevent necrotic cell death in *C. elegans*, and possibly other animals, may have important implications for host resistance to infection mediated by multiple pathogens.

## Materials and methods

### *C. elegans *strains and culture conditions

The following strains were obtained from the *C. elegans *Genetics Center (Minneapolis, MN, USA): N2 wild-type, DA531 *eat-1(ad427)*, DA465 *eat-2(ad465)*, NU3 *dbl-1(nk3) *and KU25 *pmk-1(km25)*. BC14225 *asp-5*::GFP was obtained from the Genome BC *C. elegans *Gene Expression Consortium (Vancouver, British Columbia, Canada). The *vha-12(n2915) *mutant strain was a kind gift from Erik Jorgensen (University of Utah). The *unc-32*(*e189*) mutant and the transgenic strains containing full length GFP reporters, *asp-3*::GFP and *asp-4*::GFP, have been described previously [17,32]. We generated *F44A2.3*::GFP, *vha-12(n2915)*;*asp-4*::GFP, *pmk-1(km25);asp-4*::GFP and *dbl-1(nk3);asp-4*::GFP using conventional genetic techniques. Growth and manipulation of *C. elegans *were as previously described [58,59].

### Bacterial strains and culture

Bacterial strains included *E. coli *OP50, *E. faecalis *OG1RF, *S. marcescens *Db11, *E. carotovora *CFBP 2141 and *P. luminescens *Hb. Liquid cultures of *E. coli*, *E. carotovora*, *P. luminescens *and *S. marcescens *were grown in LB, *E. faecalis *in BHI. We spread 50-150 μl of overnight bacterial liquid culture (concentrated 10-fold), depending on size of the assay plate (35 or 90 mm diameter), onto fresh NGM agar plates and incubated them for 24 h. *E. carotovora *and *P. luminescens *were cultured at 30°C, *E. coli*, *S. marcescens *and *E. faecalis *at 37°C. We used 90 mm plates for microarray and qRT-PCR related experiments, 35 mm plates for all other experiments.

### Growing worms and infection

For microarray and qRT-PCR related experiments, eggs from N2 worms suspended in M9 buffer (3 g KH_2_PO_4_, 6 g Na_2_HPO_4 _and 5 g NaCl, dissolved in 1 mM MgSO_4_) were placed at 25°C and allowed to hatch in the absence of food. Aliquots of larvae synchronized in this way were transferred after 16-20 h to NGM plates spread with OP50 and cultivated at 25°C until the mid-L4 stage. Worms were then transferred to assay plates. After 24 h at 25°C, the worms were collected, washed three times in M9 buffer and total RNA extracted. Three independent infections were performed.

### RNA sample preparation and microarrays

We added 1:10 volumes of Trizol (Invitrogen; Carlsbad, California, USA) to worms and total RNA extraction was carried out following the manufacturer's instructions. The RNA was quantified using Eppendorf BioPhotometer and RNA quality ascertained via electrophoresis with 1% non-dentauring, agarose gels. We have used microarrays with full genome coverage, each having 23,232 features against 20,334 unique transcripts generated using probes designed at the Genome Sequencing Center (StLouis, MO, USA). Oligo-probes were spotted on UltraGAPS™ slides (Corning; Lowell, Massachusetts, USA) according to the manufacturer's specifications at the Plateforme Transcriptome (Marseille-Nice genopole/CNRS/INRA; Sophia Antipolis, France). There were 24 microarrays used in this study (4 groups corresponding to the 4 pathogens with 6 microarrays per group). Experimental design included duplicate competitive hybridizations in which the Cy3 and Cy5 labels were swapped ('dye swap experiments'). Hybridization was done using an adapted version of a protocol from the Genomics Core Laboratory at the JDavid Gladstone Institutes (San Francisco, California, USA). Quenching and cleanup procedures were substituted with that described in ProtocolQQ07 from the QIAGEN literature-database. In brief, 5 μg of RNA was converted to double-stranded cDNA with superscript II (Invitrogen) using custom designed (dT)_24_-V primer (Sigma; St. Louis, Missouri, USA) and aminoallyl-dUTP (Sigma) nucleotide analogs. The cDNA was then cleaned using Qiagen PCR purification kit (Qiagen; Venlo, Limburg, Netherlands). Labeled cDNA probes were prepared via coupling to Cy3 or Cy5 mono-reactive dye packs (Amersham; Little Chalfont, Buckinghamshire, UK). After removal of unincorporated dyes with a Qiagen PCR purification kit, two differentially labeled probes were combined in a hybridization buffer containing 5× SSC, 0.2% SDS, 7 mM Tris-Cl, 0.2 mg/mL yeast t-RNA (Invitrogen), 0.2 mg/mL poly(A) DNA (Sigma). We used 55 μL of this cocktail on each chip and incubated them at 45°C for 16 h in a water-bath. Following hybridization, microarrays were processed according to recommended protocols for UltraGAPS™ slides. Microarrays were scanned on a SCANARRAY 4000 XL (Perkin Elmer; Waltham, Massachusetts, USA) machine and image analysis was performed using QUANTARRAY version 2.1 (Perkin Elmer). Spots with obvious blemishes were manually flagged and excluded from subsequent analyses. Global array quality was kept consistent with normalization coefficients for the fluorochrome channels controlled at <2, visualized using ArrayPlot version 3.0 [60].

### Expression data pre-processing

We used 20,257 genes on the microarrays, having signal strengths twice that of background and 'unflagged' data points in at least four out of six microarrays for each pathogen, as the base group for all subsequent analyses. All primary microarray data have been deposited at ArrayExpress, with accession number E-MEXP-766.

### Expression data analysis: identification of differentially regulated genes based on fold-change

Data generated from the microarrays was normalized using 'Per Spot and Per Chip: Intensity Dependent (Lowess) Normalization' in GeneSpring GX version 7.3 (Agilent Technologies; Santa Clara, California, USA). Differentially regulated genes for individual datasets (supplementary Table 1a in Additional data file 3) were arbitrarily identified using the uppermost 18.75th percentile of a dataset initially formed from probes having normalized, expression ratios (infected/control) >1.01 or <0.99 in at least 2/3 of microarrays per 'dye-swap' group for a total of 4/6 microarrays per dataset.

### Expression data analysis: exploratory analyses

Differentially regulated genes were used for exploratory analyses using clustering and gene class testing techniques. Clustering was performed within GeneSpring GX version 7.3. Two cumulative groups comprosed of genes up- (*n *= 254) and down-regulated (*n *= 266) by at least 2 pathogens (supplementary Table 1c in Additional data file 3) were separately clustered using Pearson correlation. Cluster merging was performed using average linkage and bootstrapping done with 100 datasets.

Gene class testing was performed using Expression Analysis Systematic Explorer (EASE). We annotated gene probes with Gene Ontology and euKaryotic Orthologous Group (KOG) information available from WormBase WS160 [61]. We also added functional information obtained from numerous *C. elegans*-related experiments [8-11,24,37-39,62-75]. Each dataset corresponding to up- or down-regulated genes after infection with a particular pathogen was individually tested. Over-represented gene classes were characterized by EASE scores, which are sliding-scale, conservative adjustments of Fisher exact probabilities (*p *< 0.05).

### Expression data analysis: statistical testing

As alternatives to inference based on fold-change, two statistical approaches were used to infer differentially regulated genes in our experiment. With the first, various tools as implemented in the software package J/MAANOVA version 1.0a were used [76]. Briefly, raw data from 18 microarrays was normalized using 'Joint Lowess intensity-spatial Lowess' transformation (6 each for *E. carotovora*, *E. faecalis *and *P. luminescens*). Normalized data were then analyzed with a variant of the 'mixed effects ANOVA model'; three components of variance were assumed in our model. Two fixed components were 'microarray-specific effect' (systematic variation on microarrays) and 'condition' (infected or control). A random component, 'biological replicate' was used to address random variation due to the three different sets of biological replicates used. Within J/MAANOVA, a *F*_*s*_-test [77] based on the James-Stein estimator [78] was used to identify genes differentially expressed between our two conditions of interest. Robustness of ANOVA data was tested using a permutation test; means were randomly permuted 500 times and test statistics were recalculated for differences between the two conditions. Agreement between ANOVA and permutation test results would indicate the robustness of the ANOVA model. False discovery rate (FDR) control adapted from algorithms discussed by Benjamin and Hochberg [79] and Storey [80] was applied to provide 95% confidence.

The second analysis was performed using tools within BRB-ArrayTools version 3.4.1 [81]. Raw data from 18 microarrays (6 each for *E. carotovora*, *E. faecalis *and *P. luminescens*) were transformed using 'Lowess intensity dependent normalization' to adjust for differences in labeling intensities of the Cy3 and Cy5 dyes. The adjusting factor varied over intensity levels [82]. Data were partitioned into two classes, one for infected animals and the other for control. Using the 'class comparison' multivariate permutation test and averaging dye-swapped experiments, we identified genes that were differentially expressed between 'infected' and 'control'. We used this test with 90% confidence so that the FDR was less than 10%. The FDR is the proportion of the list of genes claimed to be differentially expressed that are false positives. The test statistics used were random variance t-statistics for each gene [83]. Although t-statistics were used, the multivariate permutation test is non-parametric and does not require an assumption of Gaussian distributions.

### qRT-PCR measurements

cDNA was prepared from each sample using the following reverse transcription protocol. Total RNA (2.5 μg) was mixed with 2.5 μg (dT)_24_-V primer, incubated at 70°C for 10 minutes, then cooled on ice for 5 minutes. This was mixed into a cocktail, 0.5 mM dNTPs (Invitrogen), 1× First Strand Buffer (Invitrogen), 10 mM DTT (Invitrogen), 50 u RNasin (Promega; Madison, Wisconsin, USA) and 400 u SuperScript™ II (Invitrogen). Reverse transcription was carried out at 42°C for 1 h, the reaction terminated at 65°C for 10 minutes. All qRT-PCRs were carried out using SYBR^® ^PCR Master Mix (Applied Biosystems; Foster City, California, USA) according to manufacturer's specifications and analyzed on a GeneAmp^® ^5700 (Perkin Elmer). Expression data were collected as Ct values, where Ct is equal to the number of PCR cycles required to amplify a given gene from a cDNA population. Under 'infected' conditions, *C. elegans *grown on *E. coli *OP50 were exposed to pathogenic bacteria at the late-L4 stage, whereas 'control' animals were continuously cultured on *E. coli *OP50. Changes in gene expression for both infected and control animals were initially measured as ΔCt values and subsequently normalized against a control-gene: Pan-actin (left primer ccatcatgaagtgcgacattg, right primer catggttgatggggcaagag). For example, to measure up-regulation in infected animals (ΔCt(infected-control)), Ct values collected from control cDNA were subtracted from Ct values collected from infected cDNA. Thus, ΔCt(infected-control) = Ct(infected) - Ct(control). For all primer sets used in this study (see supplementary Table 5 in Additional data file 3), DNA amplification was linear in the relevant range of measurement; consequently, ΔCt = 1 corresponded to an approximate two-fold change in DNA concentration. Finally, fold change values were estimated by:

Fold change = ΔCt, where ΔCt is change in cycle number

### Reporter constructs/promoter GFP constructs

Expression patterns for several genes were examined via the use of promoter GFP constructs. Where transgenic, GFP-expressing strains were not already available, new strains were created as previously described [84]. Briefly, promoter fragments fused to GFP amplified from plasmid pPD95.75 were microinjected into N2 animals. PCR products were injected along with the p*col-12::dsRED *selection marker [85]. Transformed animals were subsequently identified by the presence of dsRED expression. All qualitative experiments with GFP-expressing strains were done using 40-100 animals transferred onto pathogen assay plates. Relevant information for primers and transgenic strains can be found in supplementary Table 5 in Additional data file 3.

### Pathogen colonization

Infected worms were assayed using a slight modification of a previously described procedure [86]. Fifty worms in a 15 ml tube were washed five times with 7 ml of M9 buffer containing 1 mM sodium azide. Worms were anesthetized by the effects of sodium azide during these washes. Consequently, loss of bacteria from within the animals was minimized whilst unwanted bacteria on external surfaces were removed. All subsequent steps remained unchanged.

### Survival assays

Synchronous populations of worms were established by allowing 20 adult hermaphrodites to lay eggs for a limited time interval (4-5 h) on NGM plates seeded with *E. coli *OP50. Progeny were grown at 20°C, through the L4 larval stage and then transferred to fresh plates with groups of 10-20 worms per plate for a total of 100-150 individuals per experiment. Survival assays were performed at 20°C on NGM plates containing either a pathogen or *E. coli *OP50. The first day of adulthood was defined as t = 0. Animals were transferred to fresh plates every two to four days thereafter and were examined daily for touch-provoked movement and pharyngeal pumping, until death occurred. We used the Prism software package (GraphPad Software Inc.; San Diego, CA, USA) to carry out statistical analyses and the log-rank (Mantel-Cox) test was used to evaluate differences between different conditions. Worms that died due to internally hatched eggs, an extruded gonad or prolonged periods spent at the edges of plates, were censored as described within Prism.

## Abbreviations

ANOVA, Analysis of Variance; BHI, brain heart infusion; BPI, bactericidal permeability-increasing; cisRED, cis-Regulatory Element Database; EASE, Expression Analysis Systematic Explorer; EGF, epidermal growth factor; FDR, false discovery rate; GFP, green fluorescence protein; LBP, lipopolysaccharide-binding protein; NGM, nematode growth medium; qRT-PCR, quantitative real time-PCR; vWF, von Willebrand factor.

## Authors' contributions

D Wong: research design, assays, data collection and analysis, manuscript production. D Bazopoulou: assays, data collection and analysis. N Pujol: data collection and analysis, manuscript production. N Tavernarakis: research design, data analysis and manuscript production. JJ Ewbank: study conception, research design, data analysis and manuscript production.

## Additional data files

The following additional data are available with the online version of this paper. Additional data file 1 contains supplementary figures. Additional data file 2 contains methods and figure legends for the supplementary figures. Additional data 3 contains detailed results for analyses presented in this study as supplementary Tables 1a-c, 2a-b, 3a-b, 4a-d and 5.

## Supplementary Material

Additional data file 1 Supplementary figures. Click here for file

Additional data file 2 Methods and figure legends for the supplementary figures. Click here for file

Additional data file 3 Detailed results for analyses presented in this study as supplementary Tables 1a-c, 2a-b, 3a-b, 4a-d and 5. Click here for file
