# Supplementary figures and images for: Genome-wide investigation reveals pathogen-specific and shared signatures in the response of Caenorhabditis elegans to infection
Source: Genome Biol. 2007 Sep 17;8(9):R194. doi: 10.1186/gb-2007-8-9-r194 (PMC2375032; doi:10.1186/gb-2007-8-9-r194)

# Supplementary Figure 1

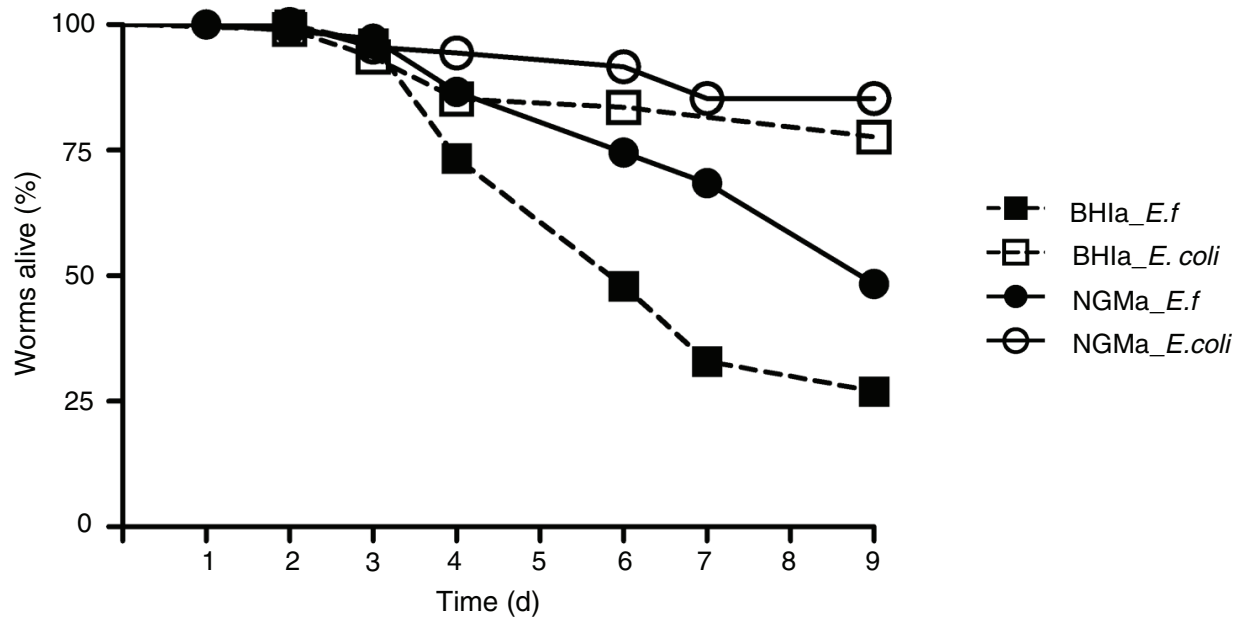

# Supplementary Figure 2

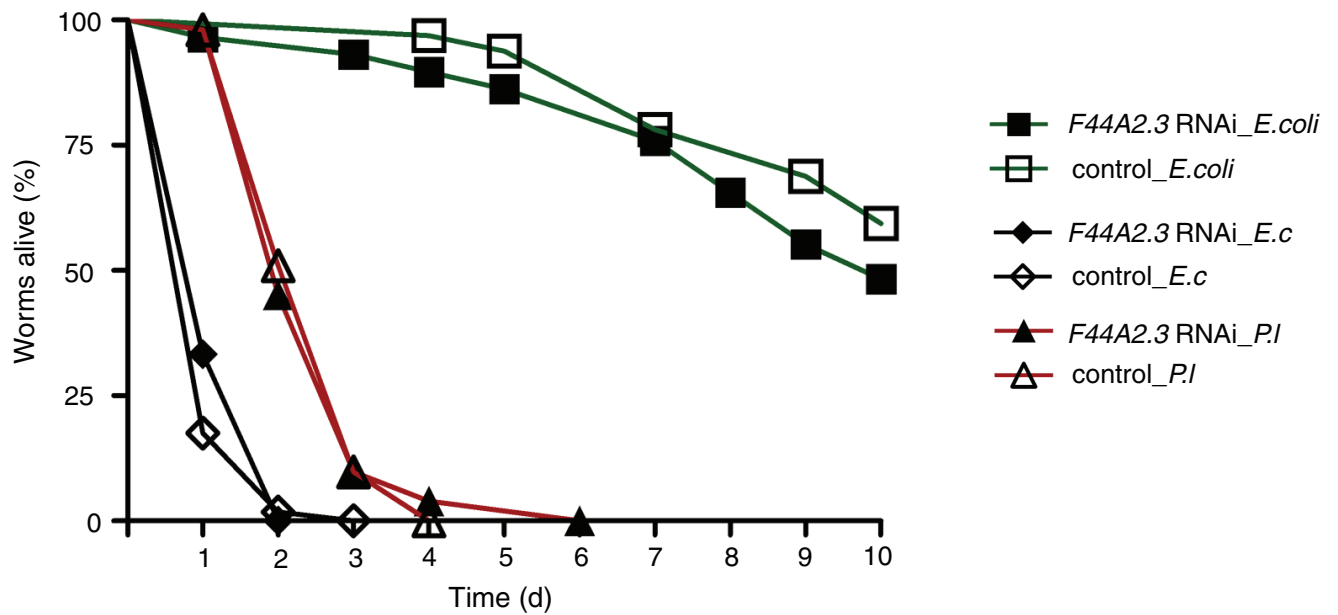

# Supplementary Figure 3

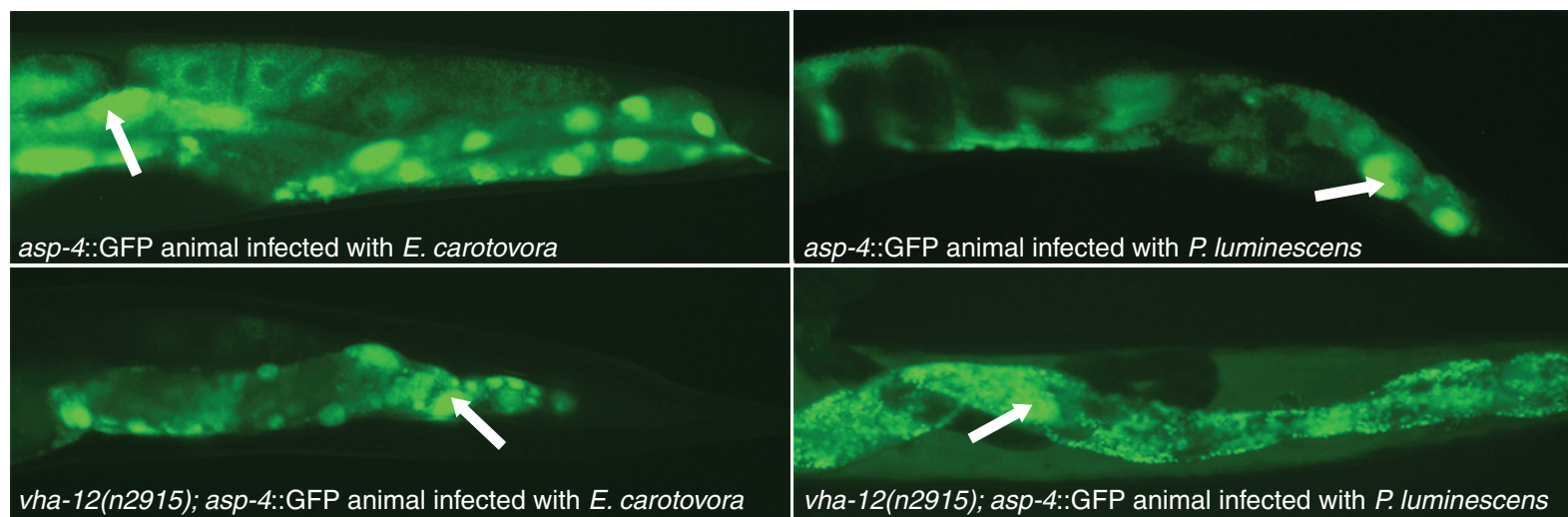

Supplement: Additional data file 1 — Supplementary figures. [file gb-2007-8-9-r194-S1.pdf]
