# Supplementary material for: Genome-wide investigation reveals pathogen-specific and shared signatures in the response of Caenorhabditis elegans to infection
Source: Genome Biol. 2007 Sep 17;8(9):R194. doi: 10.1186/gb-2007-8-9-r194 (PMC2375032; doi:10.1186/gb-2007-8-9-r194)
Supplement: Additional data file 2 — Methods and figure legends for the supplementary figures. [file gb-2007-8-9-r194-S2.pdf]

Supplementary Methods

RNAi analysis

Knockdown of *F44A2.3* expression in N2 worms, using RNAi by feeding was done as previously described (Kamath et al. 2001), using the relevant strain (Supplementary Table 5) from the RNAi feeding library (Kamath and Ahringer 2003), used after insert verification. Briefly L3 worms were placed onto RNAi plates for 24 hours at 25 °C then 50 animals were distributed equally across 5 assay plates. Experiments were carried out at 25 °C and mortality was recorded over 9 - 10 days with "dead" animals being non-responsive to touch. The Prism software package (GraphPad Software Inc., San Diego, USA) was used to carry out statistical analyses and log-rank (Mantel-Cox) tests to evaluate differences between different conditions. Worms that died due to dessication at the edges of plates were censored as described within Prism.

Survival assays with *E. faecalis* on BHI and NGM agar plates

Assay plates, of either NGM or BHI agar were prepared using liquid cultures of *E. coli* OP50 or *E. faecalis* as described in Materials and Methods. For each, 100 L4 larval staged N2 animals were distributed equally amongst 5 assay plates. These assays were performed at 25 °C and analyzed as above.

Supplementary Figure legend

Supplementary Figure 1. Survival of N2 animals on BHI (BHIa; squares) and NGM (NGMa; circles) agar plates, following infection with *E. faecalis* (infected; solid symbols) or exposure to non-pathogenic *E. coli* (control; open symbols). While the survival of control animals on BHIa and NGMa is not significantly different (Log-rank test, p-value>0.05), the differences in survival between infected and control animals, on either BHIa or NGMa are highly significant (Log-rank test, p-value<0.0001).

Supplementary Figure 2. Survival of *F44A2.3* RNAi-treated and control animals, following infection with pathogens [*E.c*:*E. carotovora* (diamonds); *P.l*:*P. luminescens* (triangles)] or exposure to non-pathogenic *E. coli* (squares) at 25 °C. The differences in

survival between control (open symbols) and RNAi treated (solid symbols) animals are not significant (Log-rank test, *p*-value>0.05). Data shown is representative of 2 independent experiments.

Supplementary Figure 3. Expression of *asp-4*::GFP upon infection in the necrosis-deficient mutant, *vha-12(n2915)*. 48 hours post-infection with either *E. carotovora* or *P. luminescens*, the expression of GFP (indicated by white arrows) in cells along the intestinal tract of *C. elegans* is similar in both *asp-4*::GFP and *vha-12(n2915)*;*asp-4*::GFP animals.
